# Supplementary material for: Inherent DNA‐binding specificities of the HIF‐1α and HIF‐2α transcription factors in chromatin
Source: EMBO Rep. 2018 Nov 14;20(1):e46401. doi: 10.15252/embr.201846401 (PMC6322389; doi:10.15252/embr.201846401)
Supplement: Supplementary file 1 — Expanded View Figures PDF [file EMBR-20-e46401-s001.pdf]

Expanded View Figures

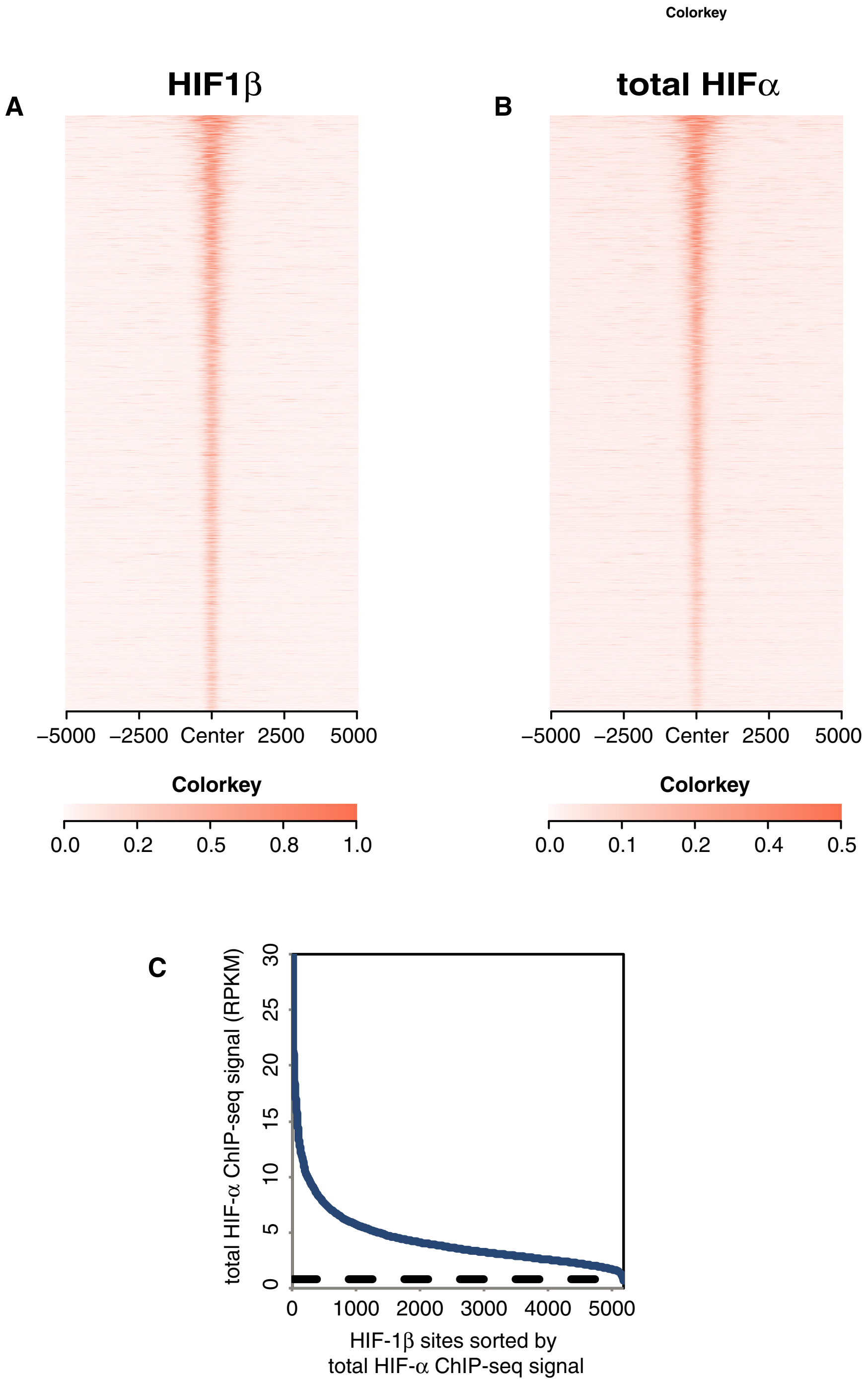

Figure EV1. Stoichiometric binding of HIF-α and HIF-1β to chromatin in HKC-8 cells incubated in 0.5% atmospheric O_2 for 16 h.

Sites that bound HIF-1β in both replicates were identified by the MACS peak caller and ordered on the *y*-axis according to HIF-1β signal intensity.

A, B Heatmaps show (A) HIF-1β signal intensity and (B) total HIF-α (HIF-1α + HIF-2α) signal intensity (read counts per million mapped reads, CPM; expressed as colour intensity, averaged across two independent ChIP-seq experiments) at HIF-1β binding sites and across the flanking ±5 kb regions (*x*-axis). HIF-α signal intensity above local background levels is observed at all HIF-1β binding sites.

C Line plot showing total HIF-α signal intensity (solid blue lines) within the MACS defined HIF-1β binding sites compared to the average background HIF-α binding signal (dashed black line) at non-HIF-1β binding accessible sites (defined by FAIRE-seq). Sites are ranked on the *x*-axis according to total HIF-α signal. HIF-α signal intensity at HIF-1β binding sites was consistently above genome-wide background levels.

 © 2018 The Authors

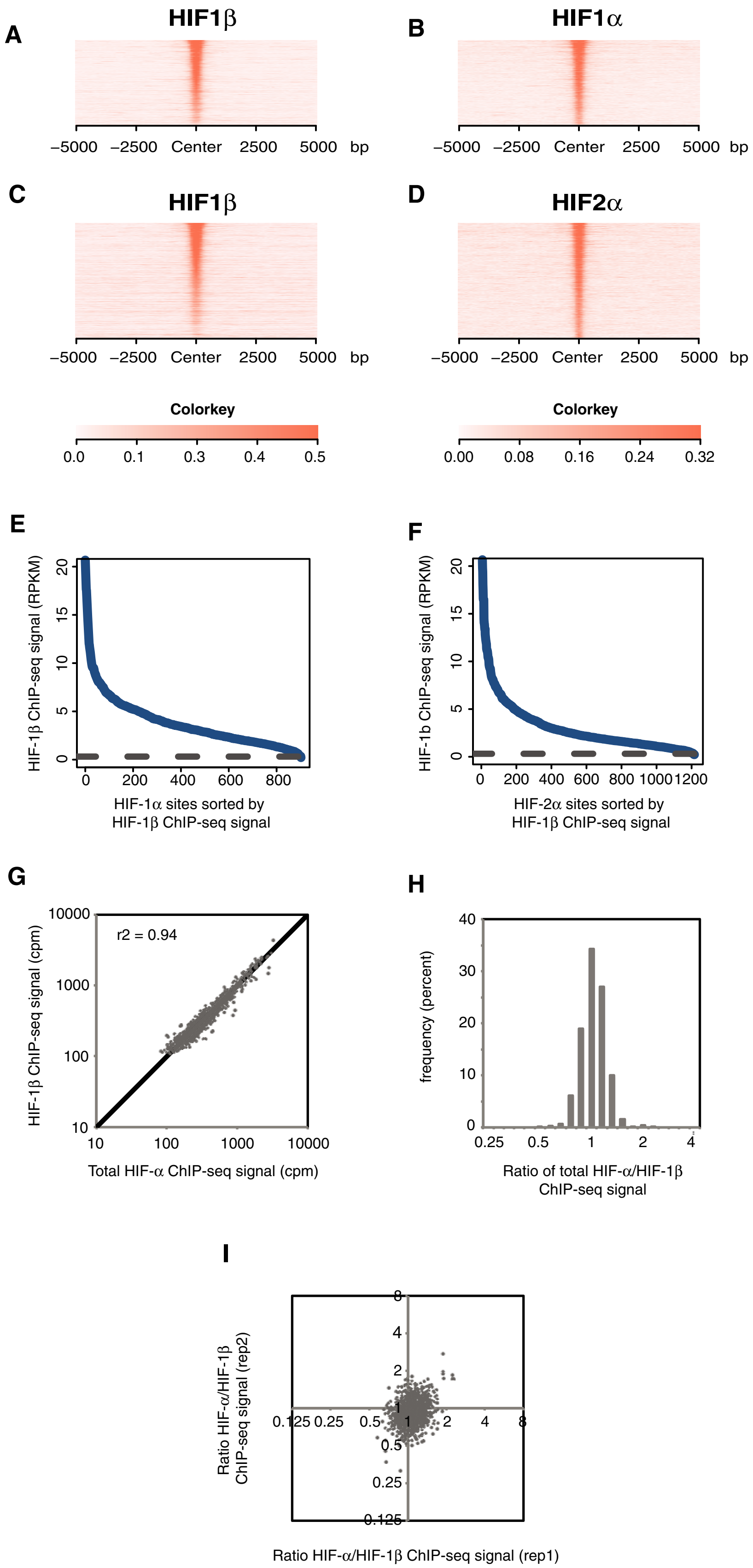

Figure EV2. Stoichiometric binding of HIF-α and HIF-1β to chromatin in RCC4 cells.

A–D Sites that bound either HIF-1α (A and B) or HIF-2α (C and D) in normoxic RCC4 cells in both replicates were identified by the MACS peak caller and ordered on the *y*-axis according to HIF-1β signal intensity. Heatmaps show HIF ChIP-seq signal (read counts per million mapped reads, CPM; expressed as colour intensity, averaged across two independent ChIP-seq experiments) at HIF binding sites and across the flanking ±5 kb regions (*x*-axis). (A) HIF-1β signal intensity and (B) HIF-1α signal intensity at HIF-1α binding sites and (C) HIF-1β signal intensity and (D) HIF-2α signal intensity at HIF-2α binding sites. HIF-1β signal intensity above local background levels is observed at all HIF-1α and HIF-2α binding sites.

E, F Line plots showing average ($n = 2$, independent ChIP-seq experiments) HIF-1β signal intensity (solid blue lines) at MACS defined (E) HIF-1α and (F) HIF-2α binding sites compared to the average background HIF-1β binding signal at non-HIF-α binding accessible sites (defined by FAIRE-seq). Sites are ranked on the *x*-axis according to HIF-1β signal. HIF-1β signal intensity at both HIF-1α and HIF-2α binding sites was consistently above genome-wide background levels.

G Total HIF-α (HIF-1α + HIF-2α) signal intensity was plotted against HIF-1β signal intensity for all sites that bound one or more HIF subunits. Strong correlation was observed between HIF-α and HIF-1β signal intensities.

H The ratio of total HIF-α signal to HIF-1β signal (*x*-axis) was determined for each site and plotted as a frequency distribution (*y*-axis) showing a tight unimodal distribution.

I The reproducibility of total HIF-α/HIF-1β ratio was assessed by plotting the ratio in replicate 1 versus that in replicate 2.

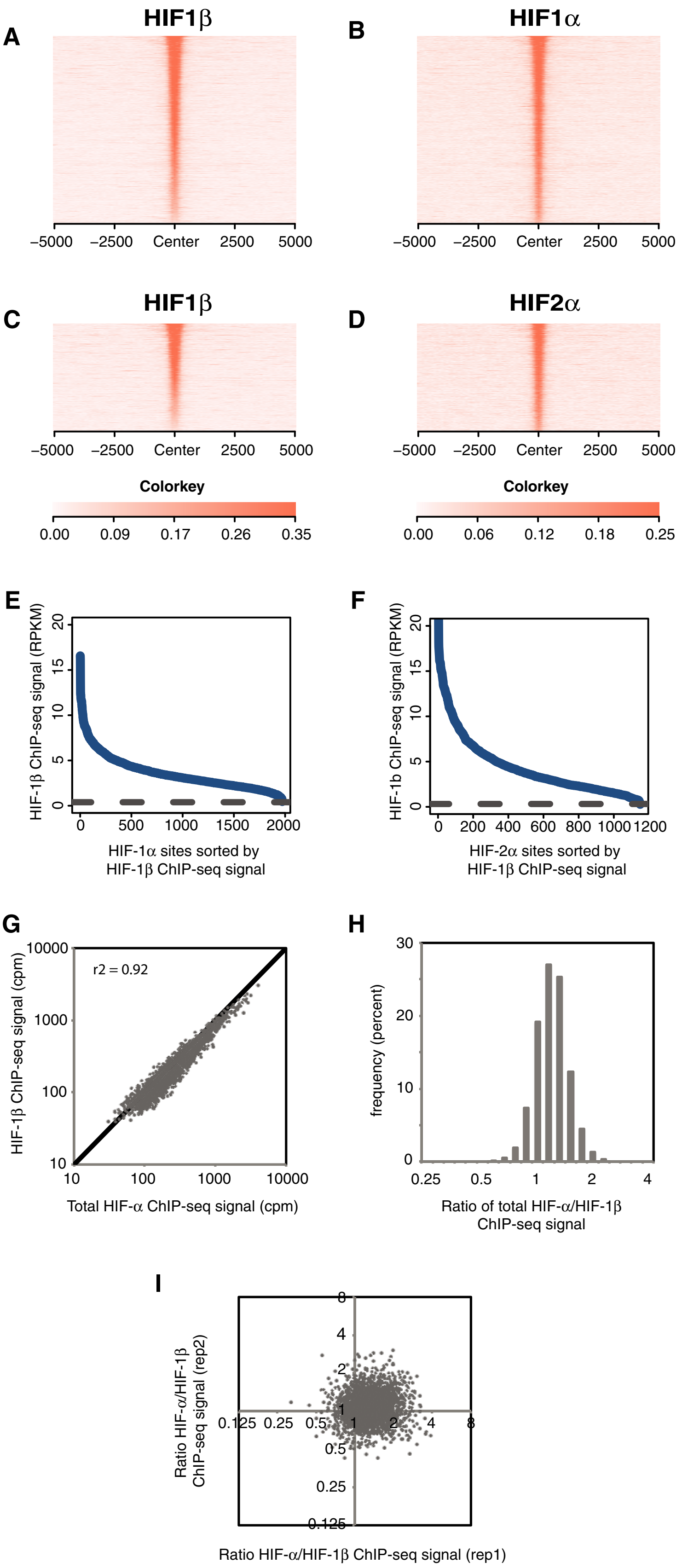

Figure EV3. Stoichiometric binding of HIF-α and HIF-1β to chromatin in HepG2 cells.

A–I As described in Fig EV2, but in HepG2 cells that had been cultured in 0.5% oxygen for 16 h.

© 2018 The Authors

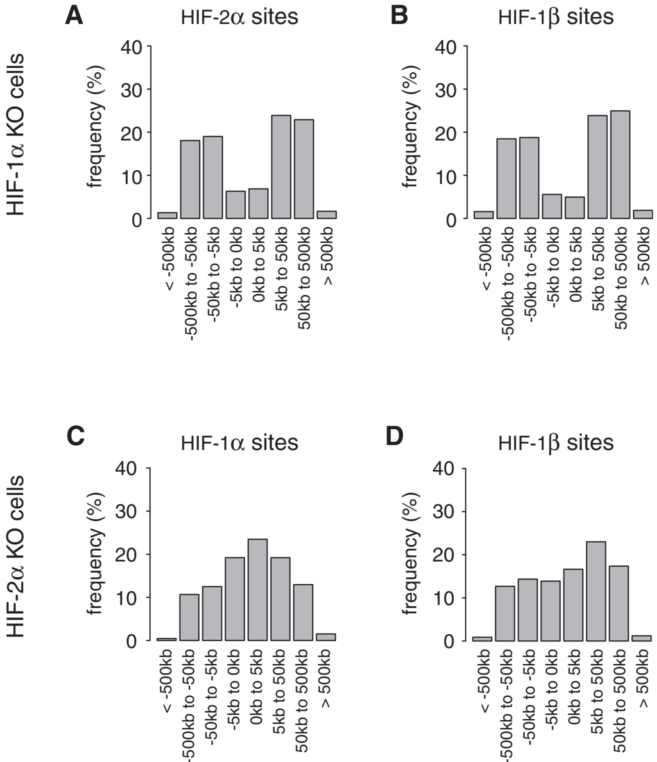

Figure EV4. Distinct binding distributions of HIF-1α and HIF-2α are mirrored by the binding distribution of HIF-1β in cells expressing only one HIF-α isoform.

A, B (A) HIF-2α and (B) HIF-1β binding sites in HKC-8 cells (present in both independent ChIP-seq analyses) in which HIF-1α had been deleted were identified using the MACS and T-PIC peak callers. HIF-1β had a promoter-distant binding distribution comparable to that of HIF-2α.

C, D The distributions of (C) HIF-1α and (D) HIF-1β binding sites in HKC-8 cells in which HIF-2α had been deleted show that the distribution of HIF-1β had changed to resemble that of HIF-1α. Cells were incubated in 0.5% oxygen for 16 h.

© 2018 The Authors

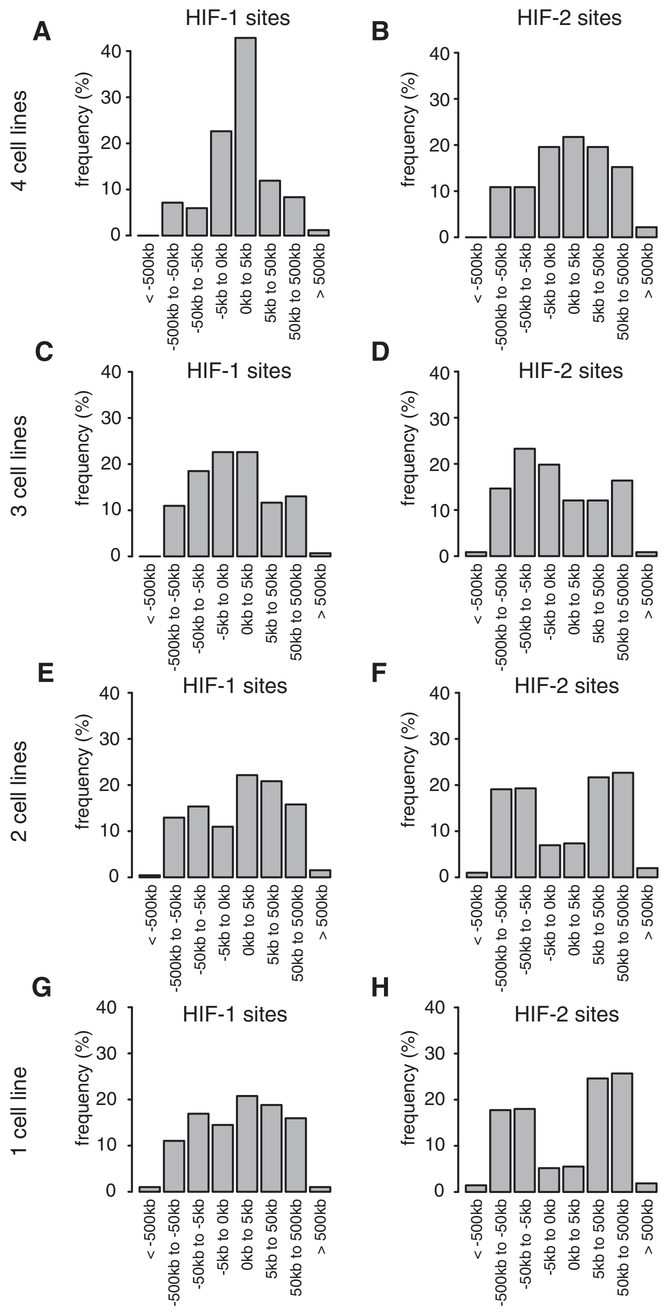

Figure EV5. Distribution of conserved and unique HIF-1 and HIF-2 binding sites about the promoter.

A–H Canonical HIF-1 (A, C, E and G) and HIF-2 (B, D, F and H) binding sites (present in both independent ChIP-seq analyses) were identified using the MACS and T-PIC peak callers. For sites conserved in four (A and B), three (C and D), two (E and F) cell lines or unique to one cell line only (G and H), the distribution of sites around the nearest promoter was plotted. The more highly conserved sites were more promoter-proximal than unique sites, particularly for HIF-1. Cells were incubated in 0.5% oxygen for 16 h.

© 2018 The Authors

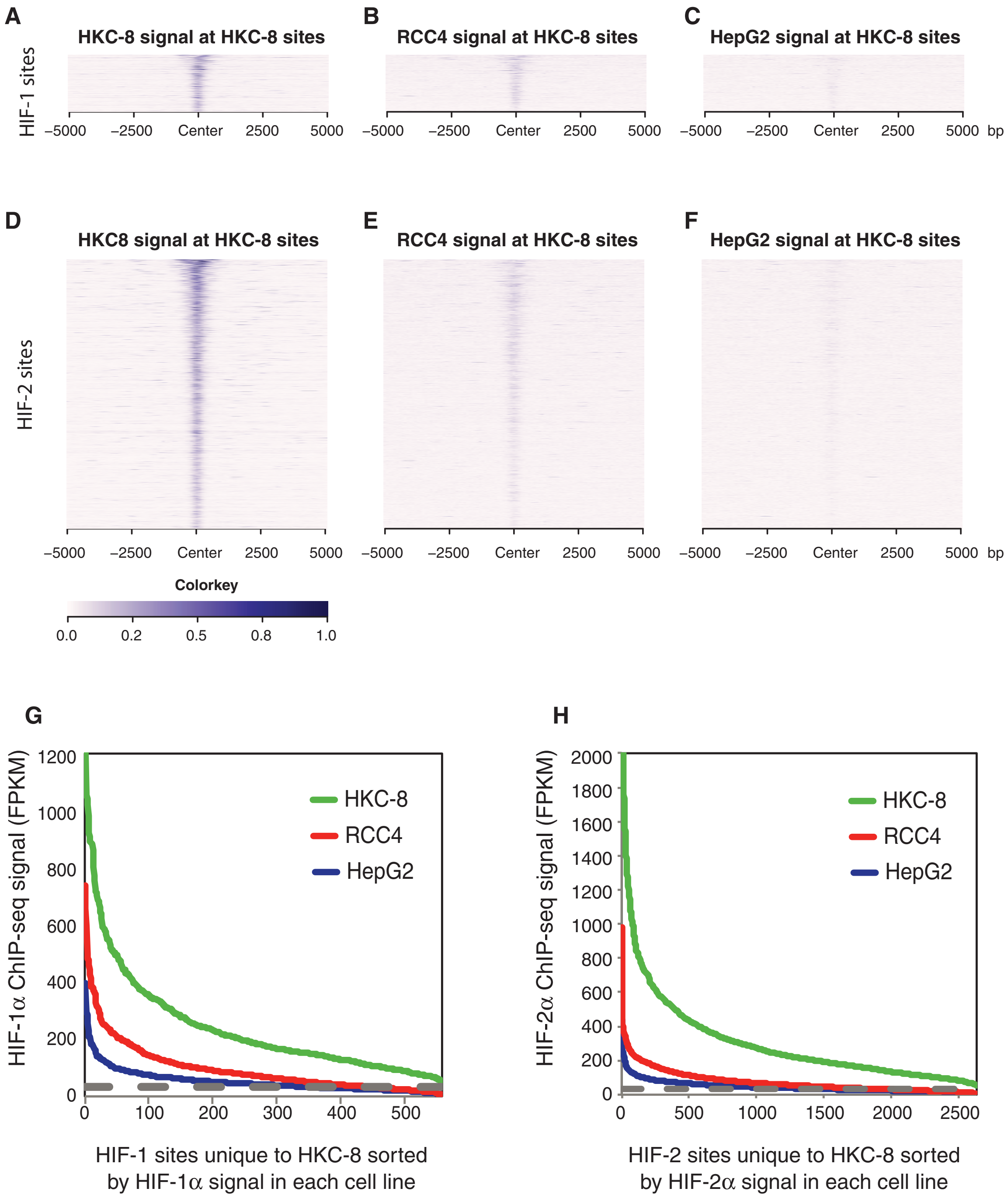

Figure EV6. Quantitative analysis of cell-type-specific HIF binding sites.

Sites that bound either HIF-1α or HIF-2α in both replicates in HKC-8 cells, but not in RCC4 or HepG2 cells, were identified by the MACS peak caller and ordered on the *y*-axis according to signal intensity in HKC-8 cells.

A–C Heatmaps show HIF-1α ChIP-seq signal (read counts per million mapped reads, CPM; expressed as colour intensity, averaged across two independent ChIP-seq experiments) at these HIF-1α sites and across the flanking ±5 kb regions (*x*-axis) in (A) HKC-8 cells, (B) RCC4 cells and (C) HepG2 cells.

D–F The same analysis for HIF-2α signal at HIF-2α sites unique to HKC-8 cells.

G Line plots showing HIF-1α signal intensity at HIF-1α binding sites identified by the MACS peak caller as binding in HKC-8 cells, but not RCC4 or HepG2 cells and ranked according to signal intensity in each individual cell type.

H The same analysis for HIF-2α signal at HIF-2α sites unique to HKC-8 cells.

© 2018 The Authors
